# Development and evaluation of the feasibility and effects on staff, patients, and families of a new tool, the Psychosocial Assessment and Communication Evaluation (PACE), to improve communication and palliative care in intensive care and during clinical uncertainty

**DOI:** 10.1186/1741-7015-11-213

**Published:** 2013-10-01

**Authors:** Irene J Higginson, Jonathan Koffman, Philip Hopkins, Wendy Prentice, Rachel Burman, Sara Leonard, Caroline Rumble, Jo Noble, Odette Dampier, William Bernal, Sue Hall, Myfanwy Morgan, Cathy Shipman

**Affiliations:** 1 Department of Palliative Care, Policy and Rehabilitation, Cicely Saunders Institute, King’s College London, School of Medicine, Bessemer Road, Denmark Hill, London SE5 9PJ, UK; 2 Intensive Care Unit, King’s College Hospital NHS Foundation Trust, London, UK; 3 Division of Health and Social Care Research, King’s College London, London, UK

**Keywords:** Palliative care, Communication, Uncertainty, Critical care unit, Intensive therapy unit, End-of-life care, Intensive care unit, Psychosocial

## Abstract

**Background:**

There are widespread concerns about communication and support for patients and families, especially when they face clinical uncertainty, a situation most marked in intensive care units (ICUs). Therefore, we aimed to develop and evaluate an interventional tool to improve communication and palliative care, using the ICU as an example of where this is difficult.

**Methods:**

Our design was a phase I-II study following the Medical Research Council Guidance for the Development and Evaluation of Complex Interventions and the (Methods of Researching End-of-life Care (MORECare) statement. In two ICUs, with over 1900 admissions annually, phase I modeled a new intervention comprising implementation training and an assessment tool. We conducted a literature review, qualitative interviews, and focus groups with 40 staff and 13 family members. This resulted in the new tool, the Psychosocial Assessment and Communication Evaluation (PACE). Phase II evaluated the feasibility and effects of PACE, using observation, record audit, and surveys of staff and family members. Qualitative data were analyzed using the framework approach. The statistical tests used on quantitative data were *t*-tests (for normally distributed characteristics), the χ^2^ or Fisher’s exact test (for non-normally distributed characteristics) and the Mann–Whitney *U*-test (for experience assessments) to compare the characteristics and experience for cases with and without PACE recorded.

**Results:**

PACE provides individualized assessments of all patients entering the ICU. It is completed within 24 to 48 hours of admission, and covers five aspects (key relationships, social details and needs, patient preferences, communication and information status, and other concerns), followed by recording of an ongoing communication evaluation. Implementation is supported by a training program with specialist palliative care. A post-implementation survey of 95 ICU staff found that 89% rated PACE assessment as very or generally useful. Of 213 family members, 165 (78%) responded to their survey, and two-thirds had PACE completed. Those for whom PACE was completed reported significantly higher satisfaction with symptom control, and the honesty and consistency of information from staff (Mann–Whitney *U*-test ranged from 616 to 1247, *P*-values ranged from 0.041 to 0.010) compared with those who did not.

**Conclusions:**

PACE is a feasible interventional tool that has the potential to improve communication, information consistency, and family perceptions of symptom control.

## Background

Communication and support for patients and their families are of central concern in healthcare, and yet too often are poorly addressed, especially at times of rapid changes in health status and in clinical uncertainty. The intensive care unit (ICU) is one setting in which patients’ circumstances can change rapidly. Although the central goal in the ICU is to preserve or extend life, the nature of illness or trauma means that more than one in five people admitted to an ICU die there [1]. Many (36 to 70%, depending on type of the ICU) deaths occurring in ICUs are preceded by a decision to limit or withhold life-sustaining treatment [1,2]. Recent initiatives, such as care pathways, have sought to improve end-of-life care, but these have focused on patients who are already known to be dying [3]. There is a need to develop a wider range of interventions before this point to improve symptom control, communication, and support in ways appropriate both for patients who may recover, and for those who deteriorate or die [4]. This is particularly important for the growing number of people who experience co-morbidities and because of the advances in medical treatments, both making prognostication ever more difficult. Palliative care is an area of healthcare focused on relieving and preventing the suffering of patients with complex needs and their families, including physical, emotional, communication, social, and spiritual aspects. Unlike much hospice care, palliative care is relevant at all stages of illness, including during curative treatment and at the end of life.

### Summary of existing literature on levels of need and interventions

We searched electronic databases to identify literature on the needs of patients and families in the ICU and potential interventions. Systematic reviews and research have highlighted concerns about support for patients and families, symptom control, communication, and decision-making, especially regarding when to stop invasive treatments, and attention to individual wishes and to dignity, respect, and peace in the ICU (Table 1) [5-25]. To address these problems, initiatives have sought to increase specialist palliative care input and/or to to embed the principles of palliative or hospice care into everyday practice [26,27]. Interventions include having a palliative care team presence at family meetings and on ward rounds, providing education and support for ICU staff, making recommendations for symptom management, and directly supporting patients and families in shared care and implementation of care pathways. However, evidence of best practice in this area is limited [18]. A randomized, controlled, feasibility study evaluating palliative care teams in an Australian ICU found no significant differences in satisfaction with care of families and staff or in length of stay, but the study was underpowered (10 patients randomized to each arm) [28]. A prospective, observational study of a structured palliative care intervention for trauma patients in the ICU [29] applied the intervention to all trauma patients because of the high mortality (15 to 20%). The study found that Do Not Resuscitate orders, life support withdrawal, and discussions of symptom management occurred significantly earlier, with no change in mortality rate, but the effect on families or patients was not assessed. The Liverpool Care Pathway for the care of the dying patient (LCP) is adapted for ICU, but needs comparative evaluation [30].

**Table 1:** Commonly identified concerns of patients and families in ICU care, in progressive illness: data from literature, phase I interviews and observation

**Areas of concern identified in the background literature review**	**Results from the family member and patient interviews, and from observation**	**Results from the staff interviews and focus groups**
Symptom management, with distressing symptoms sometimes not addressed [5-16,31]	There was a general perception among family members that symptoms were well controlled, and that timely action was taken when the patient appeared to be distressed. There was varying documentation of assessment of symptoms and symptom assessment tools changed frequently, for example, from one nursing shift to the next	Most interviewees thought that end-of-life care was well provided by the medical and nursing workforce, which was considered very experienced
Communication issues, in particular communicating the changing circumstances to patients and families; [17,31-39]	The importance of being able to ask questions and have them answered was emphasized by family members, but there was also a sense that family members felt they were responsible for getting information by asking the right questions of the right people. Particular words stood out for some people, reinforcing the need for careful choice of the words in this context. Factors described by family members as affecting communication included: their poor memory of the details of a discussion; shock affecting their ability to take in information; and their poor knowledge of medical issues making it hard for them to understand all the information given. There was an overall sense that the information given was complete and honest, that family members found it helpful if bad news could be tempered with good news, and that the uncertainty of outcome was explained well	Improved information for families including what to do in the event of a death; better privacy/side rooms for patients and family members; private space to talk to families; a position on reception for someone to greet and support family members; more support for family members and provision of on-site accommodation
Dealing with prognostic uncertainty and decision-making, which may lead to prolonged dying [25,40], or conversely, very rapid deterioration of the patient once a decision to withdraw treatment is made, with families sometimes feeling abandoned [31,33,35,36,41-44]	The greatest factor influencing involvement in decision-making was patient capacity. Preferences of family members for involvement varied; generally most of them wanted information about the process but not greater input (some preferring less). Some types of decision were more likely to be influenced by family members (place of care, aim of care, some interventions for example, tracheotomy) whereas others were more often directed by the clinician (for example, resuscitation status)	Decision-making towards the end-of-life in ICU is complex and multifactorial. Although not everyone was aware of it, the LCP was reported to be used particularly on the medical ICU. It was thought to be useful for patients on a longer dying trajectory and for those discharged to the wards, to ensure continuity of care. There were concerns about its length, but also some support because it included components of basic nursing care. It was felt by some to be unnecessary in contexts where there was one-to-one nursing care and where many patients died too quickly to benefit
Meeting individual wishes, expectations and spiritual needs that vary between different social and cultural groups [21-24]. Resource use, organ donation, and surrogate decision-making, all of which may involve conflict [21,22,33,45,46]	Family members identified factors such as financial and legal concerns; parking and transport; information needs; religious, cultural and spiritual needs assessment; and documentation and management. The importance of understanding family structures was emphasized by some family members, along with ensuring that information about the patient was given to the correct family members. When this was done well it was praised by those interviewed, but there were instances of family members feeling that their relationship was not respected by staff or that information was being given to someone other than a person designated to receive this information, and these situations caused distress	Greater support for staff and debriefing in difficult cases, although there were contrasting views about the usefulness of debriefing; a support group for nurses, particularly for those new and less experienced; better support for staff, for example the opportunity of access to an external counseling service; and more educational opportunities
Supporting dignity, respect and peace of patients and supporting the family [19]	Family members often described not knowing when discussions with staff would take place, and a few suggested that having a plan for future communications would be beneficial	Staff suggested changes to practice including: a ‘home to die’ service; more spiritual support; more hands-on nursing, massage, washing, support for family members; overt recognition of the importance of caring for the dying so that junior staff do not see it as a failure; reducing noise levels where possible; a bereavement service; and improved communication with other teams, including palliative care

Abbreviations: *ICU* intensive care unit, *LCP* Liverpool Care Pathway for the care of the dying patient.

Therefore, our study aimed to adapt or develop and evaluate a new evidence-based tool to better meet patient and family communication and holistic care needs in ICU.

## Methods

### Design and approval

The study had ethics approval from the South East London Research Ethics Committee (08/H0805/65 and 08/H808/103), and full hospital Research and Development approval.

**Figure 1:**
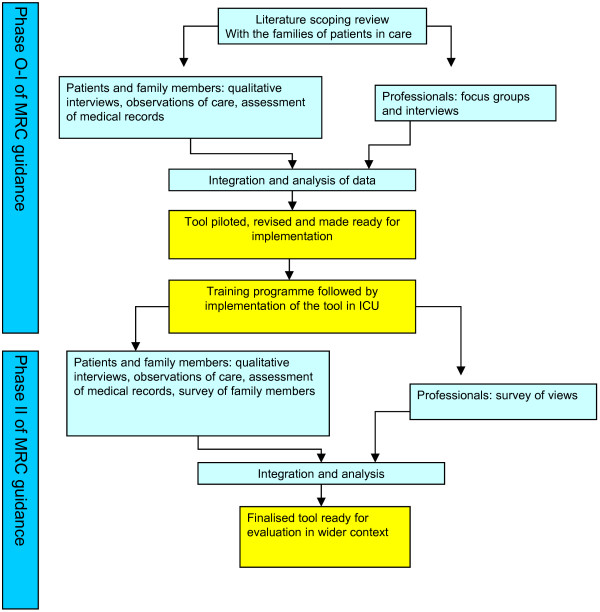
Components of the study by phase of the Medical Research Council guidance on the development and evaluation of complex interventions, showing how the different components combine to inform the next phase in the sequence.

Development, implementation, and preliminary evaluation of the tool followed the Medical Research Council guidance for the development and evaluation complex interventions [47]. Phase I explored concerns using interviews of staff and families, and observed the process of care for them, leading to tool development. Phase II tested the feasibility and effects of the tool on staff and families (see Figure 1). We used the Methods of Researching End-of-life Care (MORECare) statement of good practice to aid our procedures [48] (for compliance details, see Additional file 1).

### Setting

The study took place in two adult ICUs (general medical and surgical) in a 950-bed south London teaching hospital, serving a socially and culturally heterogeneous population. The two ICUs have 32 beds, and an annual intake of more than 1,900 admissions (1,800 excluding readmissions), associated with 250 to 300 deaths in the ICU. Most (70 the 80%) of these deaths follow life-sustaining treatment limitations. The ICU standardized mortality ratio (SMR; Knauss Acute Physiology and Chronic Health Evaluation (APACHE) II) was 0.62 during the study, and these ICUs have a lower SMR than the median SMR within the UK casemix program [49]. The ICUs service the local population and also provide support to regional trauma, neurosciences, transplantation, and cardiac services for the southeast of England. Of the admissions, 75% are white British, and 25% are non-white British (80% black Caribbean or African, and 20% from other groups). The hospital has 1,200 deaths annually (20 to 25% of deaths in the hospital occur in the ICU).

### Phase I: development and implementation of the intervention

We conducted qualitative interviews with families and staff, and observed their process of care and staff activity (see Additional file 2). Semi-structured interviews were conducted face-to-face with a lead family member (for one patient, with two family members at the family’s request), and were recorded and transcribed verbatim. A topic guide was developed from a literature review, initial observations, and discussion with service users. We conducted focus groups with ICU doctors, nurses, and other professionals involved in the delivery of care to patients and their families and palliative care practitioners to explore the experience and views of an end-of-life care pathway for the intensive care setting, using a topic guide. Staff participants were purposively selected to gain perspectives from a broad range of health care professionals, taking into account differences in age, gender, length of time working on the units, and nursing grade.

Results were presented to a working group of clinicians from ICU and palliative care, who developed the new interventional tool, termed the Psychosocial Assessment and Communication Evaluation (PACE). Initial versions were tested in three 6-week pilot periods, and amended as necessary.

#### Training and implementation

Training to support PACE implementation (Additional file 3) was followed by two further audits of the records (Figure 1). We conducted non-participant observation during and after implementation, and collected completed and de-identified copies of PACE logs. We conducted qualitative interviews with families, broadly similar in characteristics to those interviewed before PACE was introduced. These interviews explored experiences of care, and checked for any adverse effects of PACE.

#### Phase II evaluation: staff views of PACE and survey of family members

We evaluated PACE using two approaches: a survey of staff about PACE, and a survey of family members’ satisfaction with care. A staff questionnaire survey was emailed to all ICU doctors and nurses, with two reminders. The survey asked about any difficulties experienced in completing the PACE, views of its use, and its effect on care.

We attempted to survey one family member for each patient cared for on the ICU during the period of PACE implementation and use. Family members were identified and given information about the study, and then on a subsequent visit were given the questionnaire. We excluded family members whom the staff deemed were too distressed to complete the questionnaire, family members of patients admitted for less than 12 hours or family members who had visited the ICU fewer than 3 times. The questionnaire had mainly closed questions with a few open questions, and was administered by the researchers, unless the family wished to self-complete. It included the 24-item Family Satisfaction in the Intensive Care Unit questionnaire (FS-ICU) [50], validated to measure family member satisfaction with a patient’s admission in ICU. We compared responses according to whether a PACE was or was not completed for the corresponding patient.

### Analysis and sample sizes

All data were anonymized. All qualitative interviews (both phases) and focus groups were digitally audio-recorded, transcribed verbatim, and entered into NVivo9 (supplier via King’s College London, QSR International, http://www.qsrinternational.com). Qualitative data were analyzed using the framework approach [51], which enabled the systematic identification of themes both from questions and from emergent issues. This was undertaken independently by two researchers (CS, CR), with the Project Advisory Group agreeing the thematic framework. Issues (sub-themes) were then identified within these broader themes, followed by a process of charting both the patient/family and staff datasets to allow comparison between cases (specific patient care/professional group). The process of synthesis also involved mapping data from the open coding onto the broad categories identified in the preliminary literature review (Table 1).

Quantitative data were analyzed using simple descriptive statistics to describe PACE use and staff opinions. For the survey of family members we used *t*-tests (for normally distributed characteristics), χ^2^ or Fisher’s exact test (for non-normally distributed characteristics), and the Mann–Whitney *U-*test (for experience assessments) to compare the characteristics and experience of families for cases that had PACE completed with those that had not. When equality of variances was not assumed adjusted values are given (*t*, degrees of freedom, *P*, 95% CI for mean differences). All tests were two-sided.

Qualitative samples aimed to capture different groups in terms of age, gender, length of time in ICU, diagnosis, and social and cultural background, and thus capture the range of views. In the family members’ survey, most of the analysis was descriptive. However, if we assume 20% (a likely level based on previous research) of families have serious concerns, we needed 76 respondents to achieve a margin of error of 9% (95% confidence interval). Therefore, we estimated that 150 participants would provide reasonable descriptive accuracy and allow for subgroup analysis (sample stratified by age, gender, ethnicity, and disease). Allowing for refusals, we aimed to approach 200 participants. For both the qualitative and quantitative data, the samples aimed to be sufficient for preliminary exploration, as we were not formally testing PACE but to exploring its potentially beneficial or adverse effects.

## Results

### Phase I

We first interviewed 13 family members, representing 11 patients, and conducted observations on the care of these patients. Four the patients were women, age range 23 to 87 years) with diagnoses including infection, injury, and malignancy (Table 2). Observation periods for of patient care ranged from 1 to 46 days. Communication was a central theme, influencing issues such as decision-making and family support. Individualized issues of care, such as information regarding practical matters such as parking, were also important (Table 1).

**Table 2:** Demographics of family members involved in qualitative data collection in phases I and II

	**Phase 1**	**Phase 2**
Total cases, n patients (caregivers)*	11 (13)	10 (11)
Age of patients (median) years	23, 27, 31, 45, 49, 53, 54, 59, 71, 79, 87 **(53)**	40, 43,52, 57, 59, 59**,** 62, 63, 80,83 **(59)**
Gender of patients	4 women, 7 men	4 female, 6 male
Gender of family members	6 women, 7 men	9 female, 2 male
Diagnosis of patients	Neurological injury (n =4); infection (pneumonia, malaria) (n = 2); hypoxic brain injury (n = 2); malignancy (n = 2); COPD plus pneumonia (n=1)	COPD plus pneumonia (n = 2); subarachnoid hemorrhage (n = 2); C4 level lesion (n = 1); gastro-intestinal bleed (n = 1); hypoxic brain injury (n = 1); cardiac arrest secondary to PE (n = 1); acute subdural hematoma (n = 1); dissecting aortic aneurysm (n=1).
Age of interviewee **(median)** years	28,30,30, 35, 36, 44, 47, 53 ,53, 53, 56, 59,67 **(47)**	30, 36, 40, 43, 46, 48, 50, 50, 54, 62, 77 **(48)**
Relative travel time to hospital	<30 minutes (n = 4); 30 minutes to <1 hour (n = 6); ≥1 hour (n = 3);	<30 minutes (n = 2); 30 minutes to <1 hour (n = 3); ≥1 hour (n = 6 (one of these was staying locally within 30 minutes travel time));
Ethnicity of family members	White British (n = 9); Mixed Caribbean (n = 2); Asian (n = 1); Afro-Caribbean (n = 1);	White British (n = 11)
Religion of family members	No religion (n = 5); Christian (n = 6); Muslim (n = 2);	No religion (n = 4); Christian (n = 7)
Occupation of family members	Nurse (n = 2); self-employed (n = 1); civil servant (n=1); clinic supervisor (n = 1); degree-level qualification (n = 1); care manager (n = 1); lorry driver (n = 1); carer (n = 1); masters-level qualification (n = 1); technical instructor (n = 1); manager higher education (n=1); retired executive (n=1)	No paid employment (n = 4); secretary (n = 1); waitress (n = 1); carer (n = 1); security guard (n = 1); actuary (n = 1); teaching assistant (n = 1); sales consultant (n = 1)
Relationship to patient	Partner (n = 3); child (n = 6); sibling (n = 1); parent (n = 3)	Partner (n = 5); child (n = 4); sibling (n = 2)

* note in each group for one patient two family members wanted to be interviewed.

Abbreviations: *COPD* chronic obstructive pulmonary disease, *PE* pulmonary embolism.

In total, 32 staff from the ICU, 4 members of the hospital palliative care team, and 4 referring clinicians took part in individual interviews and/or focus groups, with 25 attending individual interviews, 14 attending one of three focus groups, and 7 attending both interview and focus groups (Table 3). These results also emphasized the need for psychosocial assessment and communication to be tailored to individual needs, with documentation of who had received what information when teams changed (Table 1) to improve continuity of care. However, although documentation was needed, staff wanted to ensure any tool was ‘paper-light’ and did not result in large amounts of extensive form filling. Therefore, one challenge presented in tool development was to ensure that key aspects were captured while keeping the document to one, or maximum two, pages.

**Table 3:** Staff participants in phases I and II of the study

**Staff participants**	**Phase I**	**Phase II**
Nature of data collection	Qualitative interviews and focus groups	Questionnaire survey
Medical ICU staff	6	20
Nursing ICU staff	22	67
Staff; status not given	–	5
Nursing grade	Grade 5 (n = 11; 50%); grade 6 (n = 4; 18%); grades 7 and 8 (n = 7; 32%); total: n = 22	Grade 5: 43; 65%); grade 6 (n= 16; 24%); grade 7 (n= 7; 11%); grade not given (n = 1); total: n = 67
Hospital palliative care team	4	4 (focus group)
Professions allied to medicine (ICU staff)	4	3
Referring Clinicians	4	–
Total	40	99

### The intervention: PACE

PACE draws on the work by Mosenthal *et al*. of providing a holistic assessment for all patients and families [29]. The goal of developing the tool was to improve assessment and communication for all patients in the ICU, not just those at the end of life. Thus PACE sought to improve care for patients who may deteriorate, and equally those who may recover. PACE comprises a training program and a record in the clinical notes, as detailed below.

1. PACE Training program

••One week training prior to implementation by ICU and palliative care staff - to enable all staff the opportunity to attend and to improve communication skills and awareness

••A side room on the unit housed a poster and leaflet display of relevant materials and PACE

••Members of the hospital palliative care team and researchers were present for 3–4 hours each day to introduce PACE and answer questions to foster collaboration between ICU and palliative care staff.

2. PACE record The PACE record is brief, on two sides of paper. It first asks for assessment of five aspects within 24 hours of admission to ICU:

a) Family details: key relationships; children; guardianship issues; vulnerable adults

b) Social details: financial concerns; religious/spiritual needs; language/cultural needs; transport/parking needs; other needs

c) Patient preferences: previously expressed wishes; preferred place of care; presence of advance directive / statement or will

d) Communication and information: patient / NOK awareness of situation; people to be given information; explanation of ICU

e) Any other issues: which patient, family or staff feel is important.

The next section of PACE gives space for a continuing log of any communication update.

The final section of PACE provides a list of useful resources - as a prompt to seek additional support for patients and families. Additional file 3 shows the PACE in full.

The PACE is logged by the admitting key worker, usually an ICU nurse. It is kept in the clinical records and can be updated by any member of the clinical team. The rationale for keeping PACE in the clinical record is to avoid miscommunication and improve continuity by holding psychosocial information in one place. The goal is that the assessment prompts changes in actions by staff in response to the assessment findings. The continuing log of communication enables information to flow between teams recording communication about new issues or changes in the patient or family circumstances.

PACE requires staff to explain if an aspect is not explored (e.g. staff must give a reason if it is not appropriate to discuss preferences).

### Implementation of PACE

In total, 43 nurses, 11 doctors and staff from palliative care, pharmacy, physiotherapy, occupational therapy, speech and language therapy, and chaplaincy departments attended the training during the launch week. The research process and feedback of the results led to much discussion about decision-making, communication, and end-of-life issues. In addition, there was increased input from the palliative care team on the units and psychosocial support by different providers, including chaplaincy and the palliative care team, as a consequence of the heightened awareness and identification of needs through the use of PACE.

PACE was launched in the ICU after the three piloting phases. Further revisions were undertaken on two occasions, at 6 weeks and 4 months after the original launch, to address concerns and suggestions by staff. The PACE log was usually filed in the nursing notes. An audit of the completion of PACE logs of 81 consecutive patients found that half (41) were completed within 24 hours, and the remainder within 48 hours of admission to ICU. All aspects were completed for at least some patients, the most complete being family history, next of kin, and family understanding of the condition (both completed for over 90% of cases), and social history (70% of cases).

### Phase II

#### Effect of PACE: the views of ICU staff

Responses were received from 95/176 (54%) staff members, of whom 80% (76/95) had seen a PACE log. In the main; nurses (84%; 52/62) reported completing PACE rather than doctors (17%; 3/18). Just over half (61%; 51/84) reported completing PACE within 24 hours of admission all or most of the time (similar to the audit results), but 34% (27/80) had difficulty completing some sections, especially recording patient preferences. Most (80%; 67/84) reported having seen a completed PACE in patients’ notes. The majority (89%; 71/80) reported that PACE was very or generally useful, with only 3 respondents saying it was not useful, and 6 responses being neutral. The components of PACE most often judged to be useful were family details (65 respondents), social details (52 respondents), and communication/information (25 respondents).

#### Questionnaire survey of family members

**Figure 2:**
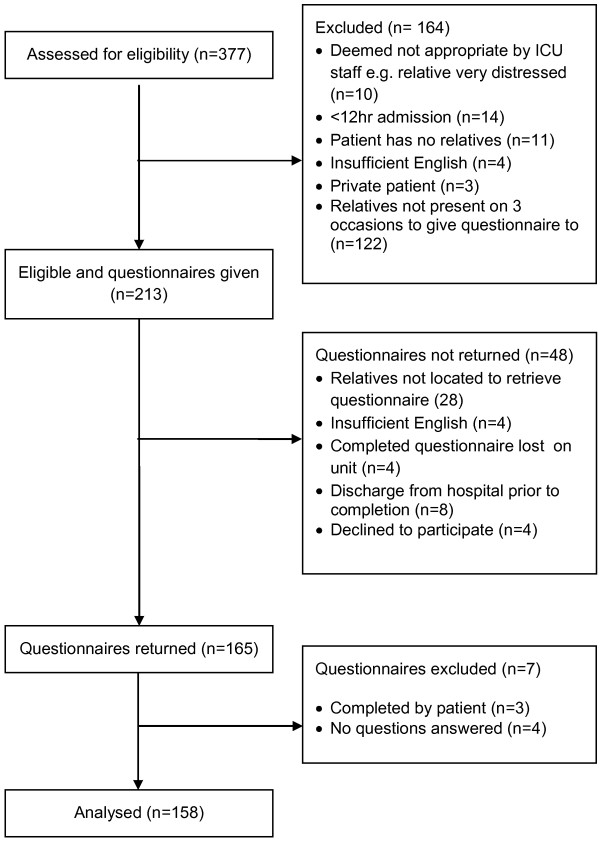
Flow chart of survey responses by family members.

Response rate from the family members was 165/213(78%). Figure 2 shows a flow chart of those eligible, and reasons for exclusion/non-response. The characteristics of the patients relating to these 165 relatives were: mean age 59 years; 48% women; 81% white, 12% black African/Caribbean/British, 5% Asian and 2% other; and had been on average 3 days on the ICU (range 1 to 56 days). We were able to compare the characteristics and experiences reported in cases when a PACE had (n = 88) and had not (n = 42) been completed (note there were instances when we could not match to PACE completion because of confidentiality and the nature of responses).

There were no differences in the demographic and clinical characteristics of relatives or patients with and without PACE completion, except that those with PACE had more severe disease and a higher risk of death (indicated by higher APACHE II scores; mean ± SD score 15.55 ± 7.22 for those with PACE completed and 13.36 ± 5.27 for those with PACE not completed, *t* = 1.95, *P* = 0.05) (see Additional file 4). Those cases for which PACE was completed had significantly better levels of satisfaction with the assessment and treatment of all symptoms (pain, breathlessness, nausea/vomiting, agitation, and confusion and communication difficulties), and for both the honesty and consistency of the information provided to relatives about the patient’s condition (Table 4). Other aspects measured also showed a trend towards improvement in the PACE group, although this did not reach statistical significance. Secondary analysis of our data did not identify cultural or ethnic differences in the reported experience or satisfaction of relatives, or the use of the PACE.

**Table 4:** **Differences in satisfaction of family members in cases when PACE was and was not completed**^**a**^

**Aspect**	**Components**	**PACE**		**Mann–Whitney *U*-test**	**PACE.** **n**	**No PACE, n**	***P*-value two-tailed**
		**Completed, median (range)**	**Not completed, median (range)**				
Symptoms	Assessment and treatment of:						
	Pain	1 (1 to 4)	1 (1 to 5)	1247.0	81	40	0.014^b^
	Breathlessness	1 (1 to 3)	1 (1 to 5)	829.5	72	31	0.014^b^
	Nausea/vomiting	1 (1 to 3)	1.5 (1 to 4)	616.0	58	28	0.032^b^
	Agitation	1 (1 to 3)	2 (1 to 4)	761.0	69	29	0.031^b^
	Confusion	1 (1 to 4)	2 (1 to 5)	655.5	63	29	0.010^b^
	Communication difficulties	1 (1 to 4)	2 (1 to 4)	698.5	64	28	0.054
Communication	Consistency of information provided to relatives about patient’s condition	2 (1 to 5)	2 (1 to 5)	1138.0	80	37	0.033^b^
	How well ICU staff informed relatives about what was happening to patients	1 (1 to 5)	2 (1 to 5)	1292.0	82	37	0.148
	Communication about patient’s condition from nurses	1 (1 to 5)	2 (1 to 4)	1352.5	81	39	0.157
	Communication about patient’s condition from doctors	2 (1 to 5)	2 (1 to 5)	1290.0	76	35	0.788
	Willingness of ICU staff to answer questions	1 (1 to 5)	2 (1 to 4)	1348.0	80	39	0.177
	ICU staff provided explanations that you understood	1 (1 to 5)	2 (1 to 5)	1315.5	81	39	0.107
Prognostic uncertainty and decision-making	Honesty of information provided to relatives about patient’s condition	1 (1 to 5)	2 (1 to 4)	1234.5	80	39	0.041^b^
	Feeling included in the decision-making process	2 (1 to 5)	2 (1 to 5)	1431.5	82	37	0.610
	Feeling supported in the decision-making process	2 (1 to 5)	2 (1 to 4)	1188.50	79	36	0.137
Meeting individual wishes	ICU staff interest in relative’s needs	1 (1 to 5)	1 (1 to 5)	1414.5	84	38	0.252
	Information about patients given to the right people (that is, those close to them)	1 (1 to 5)	2 (1 to 4)	1250.0	78	35	0.439
	Enough time to ask questions	1 (1 to 4)	2 (1 to 4)	1443.0	84	38	0.349
	ICU staff provided emotional support for relative	1 (1 to 4)	2 (1 to 5)	1090.0	75	35	0.119
	Quality of care relative and patient received in ICU	1 (1 to 4)	1 (1 to 5)	1471.0	83	40	0.232
Supporting dignity, respect, and peace	Courtesy, respect, and compassion for patient	1 (1 to 5)	1 (1 to 5)	1594.0	84	40	0.556
	Courtesy, respect, and compassion for relative	1 (1 to 4)	1 (1 to 5)	1483.0	87	39	0.179
	Care for patient from nurses	1 (1 to 5)	1 (1 to 4)	1415.0	84	39	0.110
	Care for patient from doctors	1 (1 to 4)	1 (1 to 5)	1447.0	83	39	0.264
	Atmosphere in the ICU	2 (1 to 4)	2 (1 to 4)	1583.0	82	40	0.739
	Atmosphere in the ICU waiting room	2 (1 to 5)	3 (1 to 5)	1086.0	72	31	0.824

Abbreviations: *ICU* intensive care unit, *PACE* Psychosocial Assessment and Communication Evaluation.

^a^Lower scores indicate higher satisfaction.

^b^Significant at p<0.05.

#### Qualitative interviews with families and observation post-PACE implementation

In total, 11 interviews were undertaken representing 10 patients (in 7 of these cases there was a completed PACE in the clinical records). Variation in cases between phase I and II made comparison difficult; however, the comments shed light on the quantitative results (Table 5). One interviewee (in the PACE group) highlighted the value of being able to plan a meeting with the doctor out of hours. This enabled relatives who were working to attend meetings. In both phases, preferences for involvement in decision-making varied, but relatives had a positive reaction to being asked about their preferences, even though they often did not want to be more involved in actually making the decision, as they felt this should be made by the medical staff. In one of the post-PACE instances, the palliative care social worker supported the young children of the patient visiting, promoted by the PACE tool. No adverse events related to PACE use were observed.

**Table 5:** Quotations from post-intervention qualitative interviews with family members, which helped to interpret the quantitative results

**Aspect**	**Patient comments**
Symptoms	…when he’s been on the bed he’s sort of moved and he’s gone ‘Oh’, and he said he’s in pain. I’ve then called the nurse over and automatically she’s given him painkillers, or she’s checked the chart to see when he’s had his last painkillers and given him painkillers… (Family member of patient 5, Phase 2)
	…there were reasons why it was hard to identify that actually it was… she was complaining of the abdominal pains, but because of the complications it was hard to actually sort out the factors, you know, what was the reason for it. It turned out to be the most serious of the three but that, I mean that was identified and then they flew into action… (Family member of patient 7, Phase 2)
Communication	…because I do ask… I said to them ‘is he under any sedation and have you taken him off this, have you taken him off that?’ and they’re saying about his sodium level is a bit low and I asked them what that meant and they explained that to me so, you know, they are really good… I’ve got no qualms with asking the nurses anything.. (Family member of patient 10, Phase 2)
	…some of the doctors are really good and they explain things or they say ‘do you want to talk to us?’ and then I will, I’ll ask as many questions as, you know, as I can… (Family Member of patient 1, Phase 2)
Prognostic uncertainty and decision-making	…I have to say that everybody without exception explained everything very patiently and, um, you know, could take the time to explain what was happening, what the risks were, what the likely outcomes were or could be, um, and equally to admit that on occasions they didn’t know, which was also refreshing, yeah… (Family member of patient 10, Phase 2)
	…I mean it’s been a bit sort of touch and go, um, when was it, not last weekend, the weekend before, we were told he’d only got 12 to 24 hours to live, um, and it was a bit, because it was such a shock, because he’s not had any symptoms, he’s never been ill in his life… (Family member of patient 1, Phase 2)
Meeting individual wishes	…he doesn’t want any tubes down him, he’s made that quite clear. Even if he gets worse, they were telling me his breathing, they can put a tube in to help his breathing, doesn’t want it, he said he does not want it, and his wish, you know, I said ‘Well that’s his reply, then stick to that, that’s what he wants’… (Family member of patient 5 Phase 2)
Supporting dignity, respect and peace	…they move him all the time because of sores and, you know, they’ve had to put things on his legs because his legs was turning in and, you know, when I, when I said ‘oh’, you know, and they’ve said ‘oh we put them on to straighten the leg’ and I think ‘god I wouldn’t think of things like that’. They’ve been really, really marvelous, as I said I can’t knock ‘em and I take my hats off to ‘em, and they have every time I’ve come in they’re washing and shaving him, which you don’t think of stuff like that while he’s there, d’you know what I mean? And, um, absolutely fantastic really. (Family member of patient 10, Phase 2)

## Discussion

PACE was developed and tested to help improve care in ICU, a setting in which uncertainty is common. PACE is a straightforward tool, building on the work of Mosenthal *et al*. in a trauma unit of having an approach suitable for all patients irrespective of prognosis [29], but formally integrating training, an initial assessment, and an ongoing log of communication. We found that PACE was acceptable and feasible for staff to use. The close working between palliative care and ICU staff in its development and implementation led to sustained working. It was mainly completed by nurses, which this is not unexpected, as other work has suggested the central role of nurses in ICU care [27]. Results from the survey of relatives suggest that PACE improves symptom control, communication, and information provision. An examination of the qualitative responses shed light on these results; family members were appreciative of the greater emphasis on communication and managing symptoms, which might have been supported in turn by a greater emphasis on support of the family members and of the patient’s individual needs.

### Comparison with other studies and wider implications

To embed palliative care principals and practice into ICUs, several countries and national and international associations have published recommendations, practice guides, and quality indicators [20,52-54]. Specific tools, care pathways, or bundles can also embed new approaches more formally into routine care. Some of these approaches have been used in general hospitals. Care pathways were proposed in the National Institute for Health and Clinical Excellence Guidance on Supportive and Palliative Care [55] as a possible means to improve end-of-life care, although needing more evaluation. In the USA, three before-and-after studies of care pathways suggested that there was a reduction in the number of therapies ordered, although outcomes were not studied [56-58]. Studies on the LCP in the UK found similar data using documentation and staff appraisals [59-61], and caregiver reports [60]. However, studying only processes of care rather than patient and family outcomes is flawed: Mularski *et al*. found that better documentation of pain does not in itself lead to better treatment [62]. Thus, we felt it was important, even in this preliminary evaluation, to assess outcomes, rather than the treatments received or other process measures.

In the ICU, it is difficult to accurately identify those who will die, and there is often a very short window to introduce palliative or end-of-life care and to support families. There is also a need to manage distressing and often complex symptoms, and at the same time, provide support and information to families at a time of clinical uncertainty and/or devastating news. A major problem is deciding on a trigger to use any pathway, something which has caused considerable debate in the UK with regard to the LCP [63,64]. For the ICU, integrated approaches that might benefit all patients are recommended [18], and our findings support this approach. We suggest that PACE may be suitable in cases of uncertainty in general hospital settings, possibly for all patients on admission. Recent research has shown that at the point of admission, family members feel they move from being ‘conductor’ at home, to ‘second fiddle’ where their experience and perspectives are missed [65]. Indeed, the improved communication and care at an early stage may help if any difficult decisions and conversations are needed later, at which point other tools, suitable for patients who are more likely to be deteriorating or dying, are useful.

**Figure 3:**
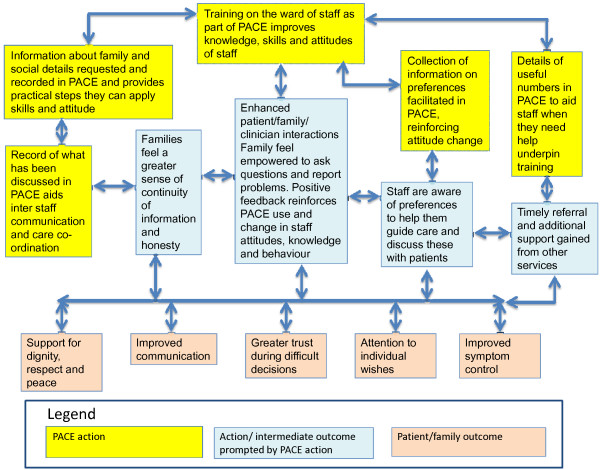
Model of how Psychosocial Assessment and Communication Evaluation (PACE) may be acting to improve care in the intensive care unit (ICU) and achieve the desired outcomes.

To underpin our results and to enable future evaluation, we developed a theoretical model of how PACE may be influencing the outcomes found in our study (Figure 3). This builds on the communication framework developed by Curtis *et al*. [66], and Nelson *et al*. [26,27] in developing models based on changing skills, attitudes, and behavior to improve communication and end-of-life care in the ICU, and the work of McCorkle *et al*. [67] in enhancing illness self-management through improved patient/clinician partnerships. As Figure 3 shows, the combination of training plus the greater individual patient/family-focused assessment in PACE (shown in yellow) can lead to improved interactions, co-ordination, and support, which in turn leads to the improved symptom control, and feelings of honesty and communication. One major goal in developing PACE was to keep the tool short and with little paperwork. Staff wanted PACE to help them do the right thing for patients and families, but not to increase documentation. Work is now underway to integrate PACE into the electronic record.

### Limitations and strengths of the study

Our study is limited by being based in two ICUs in a single hospital. However, PACE was used on a wide range of patients, with different ages, conditions, and cultural backgrounds. The findings from the surveys of staff and family members did not suggest that it was suitable for only some particular groups or circumstances. We found that PACE was used more often when patients were more critically ill, as indicated by the APACHE II score. This suggests that staff prioritized PACE for instances of greatest uncertainty. ICU wards have higher levels of staffing than general wards, and this may have supported the implementation of PACE. Therefore, wider testing of PACE in ICU units, and ideally general wards, is needed. Even if PACE is suitable only for the ICU, this represented over one-fifth of the deaths in our hospital, and so would affect a high number of patients and families.

Our comparison of family members’ satisfaction when PACE was and was not used was restricted to the ICUs participating in this study. All ICU staff had been exposed to the palliative care training and awareness engendered by PACE implementation. This may have reduced the likelihood of finding a difference between groups, because the care of all patients (including those who did not have PACE completed) was influenced. Despite this limitation, our findings of a difference between groups suggests that PACE does improve care over and above that provided by general training, although we cannot exclude the possibility that some other factor reduced the quality of care for the ‘non-PACE’ patients, which also resulted in them not receiving PACE. For example, it may have been that those staff members choosing to complete PACE were also more skilled in communication and symptom management than those who did not complete PACE. The use of PACE increased during the project, so it might also have been that care generally improved during this time. To test PACE fully, a comparative trial is required, with before-and-after assessment of care experience, including units where PACE is not used. Finally, our study was limited by the high level of satisfaction found in the survey of family members’ experiences.

## Conclusion

PACE is an acceptable and feasible tool that has the potential to improve care of ICU patients and their families. Moreover, it appears to improve communication, information-sharing and family perceptions of symptom control. We did not find any harmful effects of the tool. PACE may help the better implementation of other tools or care bundles by establishing early an assessment of the patient and family circumstances beyond medical diagnosis and improving communication. It would need to be introduced using integrated working between specialist palliative care and ICU staff, with a shared training program, and now warrants evaluation and further development in comparative trials in the ICU. PACE may also be relevant for other hospital circumstances where there is a high level of clinical uncertainty.

## Abbreviations

ICU: Intensive Care Unit; LCP: Liverpool Care Pathway for the care of the dying patient; MORECare: Methods of Researching End-of-life Care; PACE: Psychosocial Assessment and Communication Evaluation tool.

## Competing interests

All authors declare they have no competing interests; no financial relationships with any organizations that might have an interest in the submitted work in the previous 3 years; and no other relationships or activities that could appear to have influenced the submitted work.

## Authors’ contributions

IJH, WP, JK, PH, SL, WB, and MM planned the study, wrote the protocol, won funding, oversaw the study, and contributed to all components. CS and CR were appointed to work on the study, reviewed the literature, collected research data, contributed to the design, and prepared preliminary reports for the investigators. WP, RB, PH, SL, OD, JN, and WB developed the PACE based on data from phase I. WP, RB, PH, SL, OD and JN led the implementation of PACE on ICU. WP and RB led the clinical palliative care input. PH, SL, and WB provided medical ICU leadership, and OD and JN nursing ICU leadership. SH analyzed the family members’ survey. CS, CR, MM, and JK analyzed the qualitative data. IJH, WP, JK, PH, SL, WB, MM, RB, OD, and JN formed the Project Advisory Group, contributed to planning the study, the PACE tool, its implementation, study design, and the interpretation of results; approved the final report; and made substantial critical revisions of the paper. IJH drafted this manuscript and revised it in response to critical revisions from all authors. IJH and CS are the co-guarantors of the study. All authors read and approved the final manuscript.

## Pre-publication history

The pre-publication history for this paper can be accessed here:

http://www.biomedcentral.com/1741-7015/11/213/prepub

## Supplementary Material

Additional file 1 PACE study procedures according to the MORECare Statement. Click here for file

Additional file 2 Detailed description of phase I methods - Development and Modeling Leading to the Tool Development. Click here for file

Additional file 3 PACE: Psychosocial Assessment and Communication Evaluation. Click here for file

Additional file 4 Family survey responses: comparison of the patient and family characteristics where Psychosocial Assessment and Communication Evaluation (PACE) was and was not completed. Click here for file
