# Supplementary material for: Development and evaluation of the feasibility and effects on staff, patients, and families of a new tool, the Psychosocial Assessment and Communication Evaluation (PACE), to improve communication and palliative care in intensive care and during clinical uncertainty
Source: BMC Med. 2013 Oct 1;11:213. doi: 10.1186/1741-7015-11-213 (PMC3850793; doi:10.1186/1741-7015-11-213)
Supplement: Additional file 1 — PACE study procedures according to the MORECare Statement. [file 1741-7015-11-213-S1.docx]

**Additional file 1**

**PACE study procedures according to the MORECare Statement**

|  | **Recommendations** | **Response** |
| --- | --- | --- |
| Introduction/  background | 1. Present theoretical framework for the intervention and levels of need established 2. Present objectives appropriate to the level of intervention development | 🗸  🗸 |
| Study Design | 1. Indicate and justify stage in MRC guidance for development and evaluation of complex interventions, e.g. feasibility, preliminary evaluation, efficacy / cost effectiveness and wider effectiveness 2. Feasibility stages should test both feasibility of the intervention and of methods of evaluation, including outcome measurement 3. Justify methods, considering appropriate use of existing data sets and secondary analysis as these may produce rapid information 4. Justify methods of empirical studies considering mixed methods, observational studies and randomised trials | 🗸  🗸  🗸  🗸  Mixed method |
| Study team | 1. Ensure involvement from: (i) consumers, patients and caregivers; (ii) relevant clinicians; (iii) relevant methodologists to develop study questions, questionnaires and procedures; and (iv) researchers familiar with the challenges in EoLC studies 2. Ideally involvement should be well established and continuing, beyond a specific study, with joint meetings or rotations between clinical and research staff | 🗸  🗸 |
| Ethics | 1. Note in ethics committee application MORECare recommendations that it is ethically desirable for patients and families in EoLC to be offered involvement in research and MORECare evidence of patient willingness to be approached 2. Work within legal frameworks on mental capacity, consent etc, to ensure those who may benefit from interventions are offered opportunity to participate if they wish 3. Collaborate with patients and caregivers in the design of the study, vocabulary used in explaining the study, consent procedures, and any ethical aspects. 4. Attend the ethics committee meeting with a caregiver or patient, as a means to help the committee better understand the patient perspective 5. Ensure proportionality in patient and caregiver information sheets, appropriate to the study design and level of risk, as excessive information in itself can be tiring/distressing for very ill individuals | 🗸  🗸  🗸  X  🗸 |
| Participants | 1. Adjust eligibility criteria to recruit those patients who may benefit most from intervention, ensuring equipoise | 🗸 |
| Procedures | 1. Minimise burden for existing clinical staff for participation in the study 2. Clearly distinguish between service received and research activity interviews in study arms when multiple interviews with patients are undertaken in trials e.g. using a graphical system | 🗸  🗸 |
| Outcome measures | 1. Choose outcome measures that meet the following criteria:  - established validity and reliability in relevant population - responsive to change over time - capture clinically important data - easy to administer and interpret (e.g. short and with low level of complexity) - applicable across care settings to capture change in outcomes by location (e.g. patients' home, hospital, hospice) - able to be integrated into clinical care - minimise problems of response shift (see below)  1. Consider including patients' experience of care, as this is central to many interventions 2. Select time points of outcome measurement to balance the value of early recording, to reduce attrition, but to allow enough time for the intervention to have had an effect 3. Consider the potential effect of response shift (i.e. a change in a person’s internal conceptualisation or calibration of the aspects measured). Questionnaires that include anchor points or descriptions of each response category may be less problematic in this regard | 🗸  Family experience and observations  Captured  Timed before and after intervention. |
| Missing data and attrition considerations | 1. Estimate in advance levels of and reasons for attrition and missing data, integrating these into sample size estimates and planned collection of data from proxies 2. Monitor during the study and report all levels of and reasons for attrition and other missing data 3. Assume missing quantitative data NOT to be at random unless proven otherwise 4. Test results from different methods of imputation – noting that ‘using only complete cases’ is a form of imputation 5. Use the MORECARE classification of attrition to describe causes of attrition: i.e..  - ADD – attrition due to death; - ADI - attrition due to illness; - AaR - attrition at random.  1. Consider reasons for missing data which are not due to attrition. E.g. missed questionnaire, or missed data item in questionnaire. Consider these in analysis and the potential imputations | 🗸  🗸  🗸  N/A at this stage |
| Mixed method studies | 1. Mixed methods can be appropriate in all phases of development and evaluation 2. Ensure appropriate multi-disciplinary skills mix or training of team 3. Define the theoretical paradigm and method of integrating results and safeguards to ensure rigour at the outset 4. Plan investigation to avoid undue burden of qualitative and quantitative questionnaires – perhaps dividing data collection or selecting questions and/or sampling appropriately 5. Take into account any potential therapeutic effect of qualitative interviews where participants can express their feelings, if these are similar to components of the intervention 6. Ensure those collecting data are appropriately trained in qualitative data collection | Mixed method  🗸  🗸  🗸  🗸  🗸 |
| Implementation | 1. Consider implementation implications, including workforce and training needs, in all phases of the study | 🗸 model of action developed |
| Cost-effectiveness | 1. Integrate into preliminary evaluations and test feasibility of methods 2. Collect data on use of services including health, voluntary, social and informal care, to take societal approach to care costs 3. Justify appropriate outcome measures to generate cost effectiveness | 🗸  N/A at this phase  N/A at this phase |
