# Supplementary material for: Development and evaluation of the feasibility and effects on staff, patients, and families of a new tool, the Psychosocial Assessment and Communication Evaluation (PACE), to improve communication and palliative care in intensive care and during clinical uncertainty
Source: BMC Med. 2013 Oct 1;11:213. doi: 10.1186/1741-7015-11-213 (PMC3850793; doi:10.1186/1741-7015-11-213)
Supplement: Additional file 2 — Detailed description of phase I methods - Development and Modeling Leading to the Tool Development. [file 1741-7015-11-213-S2.docx]

**Additional file 2**

**Detailed description of phase I methods - Development and Modeling Leading to the Tool Development**

**Interviews were collected with**

1. ***Families***

We conducted individual face to face interviews with family members (and close friends/partners) of patients, currently severely ill on ICU and those of previous patients, including recently bereaved family members.

Inclusion criteria were: adults cared for in ICU in who there were end of life issues or discussions during ICU admission. Sample selection was purposive to achieve a range of demographic and clinical backgrounds by age, diagnosis and culture. We initially planned to interview patients, but exploration suggested that most would be too unwell for this.

1. ***Staff***

We interviewed a purposefully selected sample ICU doctors, nurses and other professionals involved in the delivery of care to patients and their families, and palliative care practitioners, to include differences in gender, age, length of time working on the units and grade. We also conducted three focus groups with staff, to further explore the main themes from individual interviews and potential solutions.

***Data collection***

Data collection comprised face-to-face semi-structured interviews, audio recorded, and qualitative non-participant observation. The semi-structured interviews among consenting family members or people close to the patient were conducted in a quiet room at a time that suited the participant. The topic guide for patient interviews was developed from reviewing the literature and in collaboration with service users. This explored, but was not limited to, their views of communication, information and symptom management, expectations of care while on the ICU, preferences regarding decision making, and perceptions of care provided to the patient and their family. Topic guides for staff interviews and focus groups explored their perceptions of issues relating to provision of care on the ICU, including communication, information and symptom management, wishes regarding decision making, experiences of care for the family and the patient, effects on staff, recommendations for improvement and views about existing tools and care pathways, including the most recent version of the Liverpool Care Pathway developed for ICU.[30]

***Observation***

Qualitative non-participant observation involved attending ward rounds and case meetings at different times of the day. We observed general activity and case specific observations (where patients/family members had consented) and continued until the patient’s death, discharge or no further end of life issues were apparent. Observations were accompanied by informal discussion with staff to clarify issues. Following consent from patient/family, medical records were reviewed using a data recording sheet to obtain relevant information on decision making or end of life issues.
