# Supplementary material for: Development and evaluation of the feasibility and effects on staff, patients, and families of a new tool, the Psychosocial Assessment and Communication Evaluation (PACE), to improve communication and palliative care in intensive care and during clinical uncertainty
Source: BMC Med. 2013 Oct 1;11:213. doi: 10.1186/1741-7015-11-213 (PMC3850793; doi:10.1186/1741-7015-11-213)
Supplement: Additional file 3 — PACE: Psychosocial Assessment and Communication Evaluation. [file 1741-7015-11-213-S3.docx]

**Additional file 3**

**PACE: Psychosocial Assessment and Communication Evaluation**

***Any member of the MDT to commence within 24 hours of admission and continue use until discharge***

**Patient name: ­­­­­________________________ DOB: _____________ Hospital Number: ___________________**

**Date / time of admission: ___________/____________** **Date / time form completed: __________/_________**

**Staff member completing form (sign & print): _______________________________**

**Family member completing form: _________________________**

**Key family contact: __________________**

**1. Family details including key relationships:**

If yes to any of the following, detail action taken below:

Children under 18? 🞏 Yes 🞏 No If yes, contact palliative care social

Guardianship issues of any children? 🞏 Yes 🞏 No worker to discuss supported visits

Vulnerable adults? 🞏 Yes 🞏 No (page KH6081)

**Action taken:**

**2. Social details** (incl. employment; religious, spiritual & cultural needs; perceptions of hospital/ ICU):

Financial concerns? 🞏 Yes 🞏 No

Religious / spiritual needs? 🞏 Yes 🞏 No

Language / cultural needs? 🞏 Yes 🞏 No

Transport / parking needs? 🞏 Yes 🞏 No

Other supportive needs? 🞏 Yes 🞏 No

**Action taken:**

**3. Patient Preferences**

**Has the patient previously expressed views** about any treatment / care wishes: 🞏 Yes 🞏 No

Specify: ____________________________________________________________________________________

Has the patient expressed a preference for place of care? 🞏 Yes 🞏 No

Specify: ____________________________________________________________________________________

Does the patient have an advance directive / statement? 🞏 Yes 🞏 No

Details and action taken: ____________________________________________________________________________________

Does the patient have a will?

🞏 Yes 🞏 No 🞏 Not appropriate to discuss currently (must give reason)

________________________________________________________________________________________________________

________________________________________________________________

(NB staff cannot witness signing of wills - for advice contact Consultant or palliative care team social worker)

**4. Communication and information:**

Is the **patient** aware of the current situation and likely outcome? 🞏 Yes 🞏 No, alert

🞏 No, conscious level

Is the **NOK** aware of the current situation and likely outcome? 🞏 Yes 🞏 No

Details and **action** taken: _______________________________________________________________________

____________________________________________________________________________________________

Names of people information about patient to be given to: _____________________________________________

Has the ITU been explained to the patient OK?

- - 1. Visiting hours 🞏 Yes 🞏 No
    2. Who to ask for information 🞏 Yes 🞏 No
    3. Who the different staff members are 🞏 Yes 🞏 No
    4. Has the relative information leaflet been given? 🞏 Yes 🞏 No

**5. Any other concerns / issues:**

**Action taken:**

**6. Communication Update** - please complete each time the patient/ NOK/other is updated

| **Date** | **Update given by** | | **Update given to** | **Communication documented?** | | |
| --- | --- | --- | --- | --- | --- | --- |
|  | Name | Designation |  | Yes/No | Medical notes | Nursing evaluation |
|  |  |  |  |  |  |  |
|  |  |  |  |  |  |  |
|  |  |  |  |  |  |  |
|  |  |  |  |  |  |  |
|  |  |  |  |  |  |  |
|  |  |  |  |  |  |  |
|  |  |  |  |  |  |  |

**Useful Contact Numbers**

**Palliative Care Team** 4060 (For 24 hour advice contact through switchboard)

**Palliative Care Social Worker** Page**:** KH6081

**Safeguarding: Adults**1773 (co-ordinator page: KH3015)

**Elderly** 6056

**Children** 1187

**Hospital social work team** 6276 / 6458 (Fax number: 6258 / 6348 or refer via EPR)

**Social work team for elderly** 6089

**Counselling service** See Nurse-in-charge to fax referral

**Chaplaincy Services** 3522 (For emergency 24hr chaplain contact switchboard)

**PALS** 3635/ 3601

**Out of hours Emergency Social Services in Southwark** 020 7525 5000 (via Town Hall switch)

**Lambeth** 020 7926 1000 (via Town Hall switch)

**Macmillan Information and Support Centre** 020 3299 5229

**For admin use only**

Code: Unit: APACHE II: Outcome: Diagnosis:
