# Supplementary material for: Development and evaluation of the feasibility and effects on staff, patients, and families of a new tool, the Psychosocial Assessment and Communication Evaluation (PACE), to improve communication and palliative care in intensive care and during clinical uncertainty
Source: BMC Med. 2013 Oct 1;11:213. doi: 10.1186/1741-7015-11-213 (PMC3850793; doi:10.1186/1741-7015-11-213)
Supplement: Additional file 4 — Family survey responses: comparison of the patient and family characteristics where Psychosocial Assessment and Communication Evaluation (PACE) was and was not completed. [file 1741-7015-11-213-S4.docx]

**Additional file 4. Family survey responses: comparison of the patient and family characteristics where PACE was and wasn’t completed.**

| Characteristic | PACE  Completed  N = 88  frequencies | PACE  not completed  N = 42  frequencies | Test coefficient P value |
| --- | --- | --- | --- |
| ICU MCCU  SCCU | 32  56 | 15  27 | χ^2^=0.00 1.00 |
| Patient’s gender: Male  Female  Missing | 43  41  4 | 22  17  3 | χ^2^=0.12 0.73 |
| Patient’s ethnicity: White  Black – African/ Caribbean/British  Other  Missing | 65  11  7  5 | 30  4  3  5 | Fisher’s Exact  0.17 1.00 |
| Patient’s religion: Christian  Other  None  Missing | 60  7  17  4 | 24  4  8  6 | χ^2^=0.34 0.84 |
| Patient’s first language: English  Other  Missing | 74  10  4 | 31  6  5 | χ^2^=0.13 0.72 |
| Family member’s gender: Male  Female  Missing | 32  54  2 | 17  22  3 | χ^2^=0.23 0.63 |
| Family member’s ethnicity: White  Black – African/ Caribbean/British  Other  Missing | 65  11  10  2 | 30  4  4  4 | Fisher’s Exact  0.17 1.00 |
| Family member’s religion: Christian  Other  None  Missing | 54  6  25  3 | 22  3  12  5 | χ^2^=0.19 0.91 |
| Family member’s first language: English  Other  Missing | 76  9  3 | 33  5  4 | χ^2^=0.01 0.91 |
| Relationship Family & patient: Spouse/partner  Other | 29  57 | 20  19 | χ^2^=3.47 0.06 |
| Family ’s previous ITU Yes  Experience: No  Missing | 29  57  2 | 7  29  6 | χ^2^=1.85 0.17 |
| Family lives with patient: Yes  No  Missing | 40  46  2 | 24  13  5 | χ^2^=3.49 0.06 |
| Family and patient contact: More than weekly  Weekly  Less than weekly Missing | 26  9  10  43 | 10  2  2  28 | Fisher’s Exact  0.69 0.76 |
| Travel time from Family ’s Less than 30 m  home to hospital: 30 m – 1 h  1h – 2 h  2 h – 3 h  More than 3 h  Close temp. accom  Missing | 19  25  25  12  4  1  2 | 11  17  5  1  1  2  5 | Fisher’s Exact  10.51 **0.05** |
|  | Mean (SD) | Mean (SD) | t p value |
| APACHE II score | 15.55 (7.22) | 13.36 (5.27) | 1.95 0.05 |
| Age of patient | 55.93 (18.75) | 62.02 (19.34 | 1.72 0.09 |
| Age of Family | 49.30 (15.65) | 49.87 (17.28) | 0.18 0.86 |
